# Stability Phenomena Associated with the Development of Polymer-Based Nanopesticides

**DOI:** 10.1155/2022/5766199

**Published:** 2022-04-25

**Authors:** María Luisa Del Prado-Audelo, Sergio Alberto Bernal-Chávez, Stephany Celeste Gutiérrez-Ruíz, Hector Hernández-Parra, Iván García Kerdan, Juan Manuel Reyna-González, Javad Sharifi-Rad, Gerardo Leyva-Gómez

**Affiliations:** ^1^Tecnologico de Monterrey, Escuela de Ingeniería y Ciencias, Campus Ciudad de México, C. Puente 222, Ciudad de México, Mexico; ^2^Department of Chemical-Biological Sciences of the School of Sciences at the University of the Americas Puebla, Puebla, Mexico; ^3^Department of Pharmacy, School of Chemistry, National Autonomous University of Mexico, Mexico City, Mexico; ^4^Facultad de Medicina, Universidad del Azuay, Cuenca, Ecuador

## Abstract

Pesticides have been used in agricultural activity for decades because they represent the first defense against pathogens, harmful insects, and parasitic weeds. Conventional pesticides are commonly employed at high dosages to prevent their loss and degradation, guaranteeing effectiveness; however, this results in a large waste of resources and significant environmental pollution. In this regard, the search for biocompatible, biodegradable, and responsive materials has received greater attention in the last years to achieve the obtention of an efficient and green pesticide formulation. Nanotechnology is a useful tool to design and develop “nanopesticides” that limit pest degradation and ensure a controlled release using a lower concentration than the conventional methods. Besides different types of nanoparticles, polymeric nanocarriers represent the most promising group of nanomaterials to improve the agrochemicals' sustainability due to polymers' intrinsic properties. Polymeric nanoparticles are biocompatible, biodegradable, and suitable for chemical surface modification, making them attractive for pesticide delivery. This review summarizes the current use of synthetic and natural polymer-based nanopesticides, discussing their characteristics and their most common design shapes. Furthermore, we approached the instability phenomena in polymer-based nanopesticides and strategies to avoid it. Finally, we discussed the environmental risks and future challenges of polymeric nanopesticides to present a comprehensive analysis of this type of nanosystem.

## 1. Introduction

Pests such as pathogens, harmful insects, or parasitic weeds represent a critical problem in agriculture because they compromise production and food guarantee. Pesticides are essential for soil protection, pest and insect elimination, and ensuring food security [1, 2]. The pesticides include insecticides, fungicides, herbicides, and rodenticides, among others, and are employed to protect the crop from pests, enhancing the crop yield. However, they can act upon other species like birds, honeybees, or humans, causing severe side effects. In addition, the indiscriminate use of these pesticides triggers pathogen resistance, reduces nitrogen fixation, and increases pesticide accumulation in agricultural and livestock goods and water environment organisms [3]. For this reason, the search for new biocompatible and biodegradable agrochemicals based on responsive materials has increased over the last decade.

In this context, nanotechnology is an exceptional option in pesticide formulations to mitigate the problems triggered by conventional pesticide methods. Nanopesticides (NPs) involve formulations with nanosized entities and present new properties derived from size [4, 5]. The higher surface area of NPs allows suitable contact with harvest pests, making them more efficient than other methods. Furthermore, the controlled release from nanoparticles permits a precise and regulated administration route for the target pests, obtaining a “pesticide delivery system.” In addition, the possible chemical modifications at the NP surface increase their selectivity, which improves crop and grain protection [2, 6].

Nanopesticides can be classified according to different criteria. For example, they can be classified based on the chemical nature: metal-based NPs, carbon-based NPs, or polymer-based nanopesticides. In particular, polymeric nanoparticles are the most promising agrochemical release system because they have already shown satisfactory results as drug delivery platforms. This behavior is related to the intrinsic polymers' characteristics such as biocompatibility, biodegradability, and chemical structure, making them suitable for surface modifications to obtain different release profiles. Furthermore, it has been reported that polymer nanoencapsulation enhances the solubility of certain pesticides and increases their activity compared to the conventional forms of delivery. For example, in recent years, the encapsulation of herbicides such as atrazine, avermectin, and metolachlor in polymeric nanoparticles based on chitosan, lactic acid (LA), or polycaprolactone (PCL) demonstrated that the herbicides increased their activity against target plants allowing the use of lower dosages [7–9]. Moreover, due to their properties, the surface of the polymeric nanocarrier could be modified to present a specific response depending on pH or temperature, and even these modifications could enhance the foliage adhesion, improving the nanopesticide effectivity [10–12].

This article provides a comprehensive review of polymer-based NPs, including their characteristics and current applications. Additionally, the instability phenomena in polymer-based NP and strategies to avoid it are approached. Finally, the environmental risks and future challenges of polymer-based NP are discussed to present a global analysis of this type of NP.

## 2. Current Development of Polymer-Based Nanopesticides

**Figure 1:**
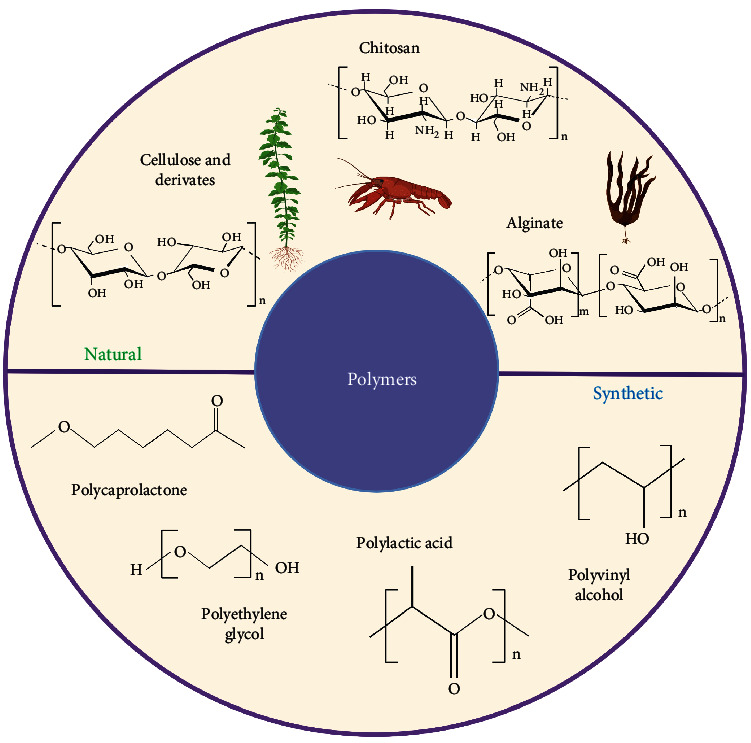
Different polymers, both from natural or synthetic sources, are employed for nanopesticides development. The figure is created with Biorender.

Despite the broad family of nanopesticides, polymeric nanocarriers represent one primary class of materials proposed to improve sustainability in agricultural operations and efficiently deliver agrochemicals such as pesticides [13, 14]. Polymer-based NPs present several beneficial properties such as biocompatibility, biodegradability, and nontoxicity [15]. Polymers can be classified based on their nature in natural or synthetic [16]. Natural polymers such as chitosan, sodium alginate, albumin, and starch or synthetic polymers like poly(lactic-co-glycolic) acid (PLGA), PCL, and polyvinyl alcohol (PVA) have been employed in the NP development [13, 17] (Figure 1). Different natural and synthetic polymer-based NPs are shown in Table 1 [18].

### 2.1. Natural Polymers

Polymers derived from natural sources are extensively employed in nanopesticide formulations due to their abundant nature and intrinsic characteristics. Examples of polymers used as nanocarriers for pesticides are chitosan, alginate, and starch.

#### 2.1.1. Chitosan

Chitosan is the main component of the exoskeleton of invertebrates and cell walls of some bacteria. This cationic polymer is obtained in the industry through partial deacetylation of chitin and consists of alternating units of *β*-(1→4)-linked-d glucosamine and N-acetyl-d-glucosamine units. It is known that chitosan is biocompatible, pH-responsive, and biodegradable, which makes it an excellent candidate as nanopesticide material. For instance, in 2020, a research group evaluated the activity of paraquat, a contact herbicide used in the control of weeds, in a nanosystem based on chitosan and tripolyphosphate [19]. The nanoherbicide exhibited a higher electroactivity than the nonencapsulated paraquat. Chitosan has also been employed as a coating for nanosystems due to its intrinsic positive charge and the functional groups in its chains, which facilitate structural modifications [20]. In 2021, Dong et al. worked to develop a pH- and temperature-responsive nanocarrier system to release paraquat [21]. For this, carboxylated porous carbon nanoparticles were employed to encapsulate the herbicide, and the nanoparticles were chitosan-coated, inhibiting the paraquat release in acidic and alkaline pH values. Similarly, Xiang et al. developed a nanopesticide system based on a magnetic carrier (diatomite/Fe_3_O_4_) coated with chitosan for controlled release of glyphosate and cypermethrin [22]. The nanostructure presented a suitable adhesion capacity in the weed surface and pest epidermis due to the chitosan presence, which enhanced the controlled release of the active substances.

#### 2.1.2. Alginate

Alginate is another polymer widely employed in nanotechnology [23, 24]. This biopolymer is usually extracted from brown seaweed, and it is composed mainly of L-guluronate and D-mannuronate residues [15]. It possesses properties such as biocompatibility, low toxicity, and affordability and presents mild gelation in the presence of divalent cations, making it a suitable candidate for NP development [25]. Alginate nanoparticles have been designed for pesticide, fertilizers, and herbicide release. In this context, nanosystems based on alginate exposed appropriate behavior for encapsulating hydrophilic herbicides such as dicamba [26]. The nanoformulation presented homogeneous size distribution and a release dominated by diffusion through 10 days, demonstrating the nanosystem's efficacy. Alginate is usually used in combination with other materials such as chitosan, silica, and cellulose, to enhance the properties and improve the application [27–30].

#### 2.1.3. Cellulose

Cellulose is the most abundant biocompatible polymer in nature that many bacteria and fungi can degrade; however, it presents a lack of water solubility. On the other hand, carboxymethyl cellulose, an anionic cellulose type, has superior solubility and remains the biological properties of cellulose. This polymer has been used in NP design, alone or mixed, displaying promising results. In 2020, cellulose nanocrystals were used to encapsulate thiamethoxam, a fast-acting systemic insecticide that belongs to the neonicotinoids group [31]. The nanocrystals, with a zeta potential value of -23 mV, were stable and presented an entrapment efficiency of thiamethoxam around 84%. Furthermore, it exhibited a sustained release and an effective insecticide activity against Phenacoccus solenopsis, even superior to the commercial formulation.

Nanocarriers based on carboxymethyl cellulose have been mixed with compounds such as rosin or diallyldimethylammonium chloride [19, 20], presenting effective leaf adherence, preventing loss, and increasing the controlled release of the active molecules. Polymers like starch and cyclodextrins (enzymatic degradation products of starch) are also suitable options to develop nanocarriers for agriculture applications. However, the most used natural polymers nowadays are chitosan, cellulose, and alginate. Besides natural polymers, synthetic polymers are an option for agricultural applications, and mixes of natural and synthetic polymers have been demonstrated appropriate behavior.

### 2.2. Synthetic Polymers

Like natural polymers, synthetic ones have to possess specific characteristics to form a suitable nanocarrier matrix. There are different examples of synthetic biodegradable polymers used in medicine and biology and are essential components of drug delivery vehicles, tissue engineering scaffolds, and biomedical devices that are also good candidates for NPs. Examples of these are PCL, polyethylene glycol (PEG), and polylactic acid (PLA) [32].

#### 2.2.1. Polycaprolactone (PCL)

PCL is a biodegradable polyester that is partially crystalline and presents a low melting point. It is prepared by ring-opening polymerization of *ε*-caprolactone using a catalyst such as stannous octanoate and is degraded by hydrolysis of its ester linkages in physiological conditions. For these reasons, this polymer has mainly been used as nanocarriers for herbicides [13, 33, 34]. For example, in 2019, pretilachlor-loaded PCL nanocapsules were evaluated as a weed control treatment [13]. The results revealed that the nanosystem was not toxic for rice (Oryza sativa), the nontarget plant; meanwhile, it was effective against the target, barnyard grass weed (Echinochloa crus-galli). Furthermore, the highly biostable system increased herbicide activity instead of the commercial pretilachlor [13].

Besides the analysis in the activity increment, the evaluation of release mechanisms from NPs is crucial due to their applications. Recently, a deep study of metribuzin release, in water and soil, from PCL nanocapsules was carried out [32]. Different concentrations of metribuzin were loaded in PCL by extrusion, observing that, in water, after 7 days of study, 96% of the herbicide was released. In contrast, only 20% of metribuzin was released after 14 weeks in soil.

#### 2.2.2. Polylactic Acid (PLA)

Likewise, PCL and PLA have been extensively investigated in molecule delivery technology for controlled release. This polymer, approved by the Food and Drug Administration, has been utilized for insecticide delivery, such as lambda-cyhalothrin, abamectin, and azoxystrobin [1, 35–37]. The research probes that using PLA as a nanocarrier for these active molecules improves their activity in active aphids (Myzus persicae L.) due to the retention rate of nanocarriers on foliage. The retention time on foliage surfaces and the wettability of the nanocarriers play a key role in the NP design. These properties could be enhanced using molecules that promote adhesion, like tannic acid or PEG [37].

#### 2.2.3. Polyethylene Glycol (PEG)

Several reports of using PEG are found in literature, both as a core material for NPs or as a functionalization material for NP surfaces [38, 39]. This versatile polyether is being utilized in various applications. For example, Fernández-Pérez et al. reported that the combination of lignin and PEG for metribuzin delivery is a suitable formulation for soil applications, exposing a high encapsulation percentage [40]. Carbofuran is another insecticide that has been entrapped in PEG nanostructures, exhibiting a sustained release between 21 and 49 days, depending on the PEG molecular weights [41].

## 3. Structure of Polymer-Based Nanopesticides

The structure of NPs can involve nanosized active substances coated with polymers, nanosized active substances stabilized with polymers on the surface, polymeric carriers mixed with lipids with trapped active substances, vectorized polymeric carriers [47], and other forms of support such as nanofibers and nanogels. Traditionally, nanoparticles can be distinguished in the nanosphere and nanocapsule classification. The nanosphere is a solid or semisolid polymeric core with the active substance molecularly dispersed or in the form of crystalline or amorphous particles. In contrast, the nanocapsule is a polymeric shell surrounding an empty core, liquid or gas-like. The several carrier variants are preferably defined by the physicochemical properties of the active substance and the method and purpose application. The application of aqueous dispersions of nanoparticles predominates in the literature due to the ease of application in wide coverage.

### 3.1. Nanocapsules

Polymeric nanocapsules are structures composed of a hydrophilic or hydrophobic internal cavity surrounded by a polymer coating [17, 48]. The active substances are generally dissolved in the internal liquid core and encapsulated by polymers spontaneously during the formation of nanocapsules [49]. This polymeric layer is responsible for the controlled release of the active principle from the core [16, 49].

Different polymers, especially preformed ones such as PLA, PCL, and polylactic coglycolic acid (PLGA), are highly biodegradable, biocompatible, and nontoxic, and their physical and chemical properties are widely known; they are used to synthesize nanocapsules [17, 50, 51]. The most used methods for the synthesis of nanocapsules are nanoprecipitation, emulsion-diffusion, emulsion-evaporation, emulsification coacervation, and layer-by-layer assembly [16, 41]. Nanoprecipitation consists of mixing bioactive molecules and the polymer in a partially soluble organic solvent, adding an aqueous solution with surfactants, and when the solvent evaporates, the nanoparticles remain in suspension. In contrast, during the emulsification-diffusion method, unlike the previous one, in the final part, water is added to the suspension to remove the solvent and precipitate the nanoparticle. The emulsion/evaporation method consists of the emulsion of two phases, one aqueous with an emulsifying agent and an organic phase immiscible in water, with polymers that precipitate encapsulating the bioactive molecules. The double emulsion method has made it possible to encapsulate hydrophilic molecules when they bind to a surfactant and dissolve in water. The “layer-by-layer” self-assembly system involves simple adsorption of oppositely charged polyelectrolytes onto core materials to create multilayer nanostructures that are held together by electrostatic forces [30, 51, 52].

The polymer composition of the encapsulated solution directly influences the encapsulation efficiency. For example, researchers synthesized PEG nanocapsules loaded with essential garlic oil and found that the optimal ratio of essential oil to PEG influenced loading efficiency. The charging efficiency reached 80% with a ratio of essential oil to PEG of 10% [53]. Therefore, the polymer composition concerning the encapsulated solution directly influences the encapsulation efficiency [16].

In general, the release of pesticides from polymer-based nanocapsules occurs by diffusion of the active compound in the nucleus through the polymeric membrane until it reaches the surface [34]. Multiple factors can interfere with the release mechanisms of nanocapsules, and it has been reported that the release rates of the active principle are directly proportional to the molecular weight of the polymer [16, 54]. In addition, polymer's mechanical properties, the degree of biodegradability, the thickness of the coating, and other factors such as the physiology and the water or cations content in the soil significantly affect the mechanism of active principle release [33, 45, 50]. In 2017, Petosa et al. evaluated the efficiency of pyrethroid bifenthrin delivery from poly(methacrylic acid-ran-butyl methacrylate)-based nanocapsules in different model soil systems compared to a commercial formulation [55]. The efficiency of transport of nanocapsules and the commercial formulation were evaluated as a function of cation species or ammonium polyphosphate fertilizer presence and sand type. The results provided an approach to understanding the interactions between the delivery system, agricultural soil, and the mechanisms that regulate the transport of these systems.

### 3.2. Nanospheres

Polymeric nanospheres are spherical structures based on a dense polymeric network in which the pesticide can be trapped inside or adsorbed on their surface [49, 56]. The polymer matrix can be amorphous or crystalline and can protect the active ingredient from enzymatic and chemical degradation [17, 18]. The polymeric matrix could be coated with surfactants or other materials that stabilize the formulation. Given the amount of polymer in the nanospheres, they can provide greater protection and a more prolonged release of the active ingredient than nanocapsules [2, 57].

The encapsulation of the active molecule in nanospheres is achieved by adding it during the nanoparticle manufacturing process. The adsorption of the pesticide or herbicide on the nanoparticle surface requires derivatization of nanoparticles (when a covalent adhesion is desired), or it simply occurs by incubating the active molecule with the nanoparticle suspension (when a reversible adhesion is desired). In 2019, Barrera-Méndez et al. encapsulated propiconazole fungicide in PLA nanospheres and evaluated their efficiency in treating the Fusarium dieback disease [58]. Based on the release profile and antifungal activity, the nanospheres presented an improved performance than the commercial form of propiconazole, revealing a sustained release during 55 h.

Nanospheres are also candidates to be modified on the surface to improve some properties, extend the release, or both. In this regard, different types of magnetic nanospheres with polymer coatings were developed to obtain two benefits: (i) the pesticide release from the polymer and (ii) the nanosphere collection at a high concentration from water or soil, taking advantage of the metallic behavior [22].

The loading method forms covalent bonds and may induce chemical modifications of the active molecule, which could or not alter its activity. Therefore, the possible active molecule-polymer interactions should be considered [59]. For example, Werdin González et al. demonstrated that besides chitosan biocompatibility is well known, chitosan-based nanospheres for geranium and bergamot release increase the toxicity of these essential oils, an effect not observed in loaded-PEG nanospheres [46]. The authors suggested that interaction between chitosan and the active ingredients may alter the toxicokinetic profile.

Although the synthesis process of nanospheres is very similar to nanocapsules, the mean particle size, size dispersion, and loading efficiency of the nanospheres depend on the polymers employed [57]. The size of the nanospheres depends, among other aspects, on the molecular weight of the polymers, observing a larger size with larger polymers (high molecular weight) [1, 60–62].

### 3.3. Nanogels

Nanogels, also known as hydrogel nanoparticles, are water-swollen nanosized polymeric networks. They consist of either physically or chemically cross-linked polymer chains (hydrophilic or amphiphilic) that can swell but do not dissolve in water [63]. The properties of nanogels can be controlled via the functional monomers, the degree of crosslinking (produced by different methods, such as ionic crosslinking, self-assembly, crystallization, crosslinking polymerization, radiation crosslinking, or functional group crosslinking), and the preparation method, to enhance responsiveness to the surrounding environment [64, 65].

For this purpose, Kala et al. [66] developed chitosan-acrylate nanogel containing lemongrass (Cymbopogon citratus) oil for durable antimosquito finishing of cotton fabric being the acrylate incorporated as a thickener and fabric binder. The bioefficacy of nanogels, postfifteen washing, was 75% of repellency against mosquitoes using acrylate, while in nanocapsules without acrylate, only 51% of repellency was achieved. The nanogel did not show any signs of dermal toxicity, which makes a suitable formulation to impregnate fabrics. On the other side, chloroinconazide-loaded alginate-based nanogel was developed to increase the efficacy and duration of pesticides. The nanogel presented higher foliar adhesion than chloroinconazide and exhibited a sustained release for up to 7 days and continuously activated the reactive oxygen species antioxidant levels, inducing the increase of salicylic acid content and the expression of its responsive gene PR2 for a long time. Thus, achieving sustained resistance to tobacco mosaic virus infection in Nicotiana benthamiana [67].

Nevertheless, despite the relevant results that nanogels can provide, a disadvantage is that many pesticides are oils with low solubility and affinity to hydrophilic materials. For this reason, the use of polymers capable of forming nanogels and allowing the encapsulation of these pesticides has increased. For example, the essential oil of Lippia origanoides Kunth has shown repellent and insecticide properties; thus, Almeida et al. [68] encapsulated the oil in nanogel chitosan modified with ferulic acid. Ferulic acid reacts with the amino groups of chitosan, improving their physicochemical properties, resulting in a material with greater lipophilic molecule affinity. The best nanogels, regarding encapsulation efficiency and essential oil stability, were obtained for the highest amount of ferulic acid.

On the other hand, Beyki et al. [65] encapsulated Mentha piperita essential oils, as alternatives for toxic synthetic fungicides, in chitosan–cinnamic acid nanogel, which enhanced antimicrobial activity against Aspergillus flavus. The cinnamic acid was used as a hydrophobic moiety to increase the oil encapsulation efficiency. Due to the volatility and instability of the oils against environmental factors, the encapsulation considerably improved its performance with a lower minimum inhibitory concentration against the free form of the Mentha piperita essential oils. The free oils failed to cause complete inhibition within the concentration range tested (up to 3000 ppm), while the encapsulated oils produced the same effect at 800 ppm when tested under nonsealed conditions.

In order to reduce the environmental impact of NPs, the group of Zhang et al. [69] developed a greener nanodelivery system made of PVA-valine or PVA-lignin, an eco-friendly polymer, loaded with emamectin benzoate through electrostatic and coacervation. The emamectin benzoate, a derivative of the avermectin family, possesses higher activity than traditional pesticides. The nanogel was prepared via a modified emulsion solvent evaporation method and presented favorable stability at low and high temperatures and in water with different hardnesses. In addition, the leaves exhibited higher retention of the nanogel than an emamectin benzoate emulsifiable concentrate. Also, the nanogel possesses higher antiphotolysis properties and bioactivity against Plutella xylostella than the emulsifiable concentrate. Another example of biopesticide nanogel created by Brunel et al. [70] was a copper (II)–chitosan nanogel, which presented suitable stability and ease of handling compared to chitosan solutions. Copper and chitosan components exhibited a strong synergistic effect in inhibiting Fusarium graminearum growth. On the other side, nanogels with stimuli-responsive properties have been developed. A lignin methacrylate-based nanogel copolymerized with acrylic acid revealed pH-responsive swelling behavior, and it was capable of releasing the chlorpyrifos pesticide in alkaline conditions [71].

### 3.4. Nanofibers

The nanofibers are a class of nanomaterials with cross-sectional diameters which possess extremely high specific surface area and surface-area-to-volume ratio. Nanofibers can form networks of highly porous mesh with remarkable interconnectivity between their pores. The nanofibers are usually synthesized by electrospinning; however, several variations of this method have been developed, including the multi-needle, needleless, and coelectrospinning or coaxial electrospinning [72]. Nanofibers can be found in natural and synthetic form and have several applications, including aerospace, 3D printing industry, orthopedic and structural applications, polyurethane matrix, paper, and textile industry [73]. In agriculture, nanofibers with encapsulated fungicides and herbicides can protect plants, improve plant growth, avoid pollution and contamination, promote irrigation systems, and detect some pesticides in water [74]. However, nanofibers could also be sustainably applied in many agricultural processes, reducing the loss in used agrochemicals, pesticides, hormones, and/or fertilizers [73].

Pesticides have been described as generally toxic and create/increase phytopathogenic organism resistance and pollute the phreatic zone. Therefore, encapsulation has been increasing to reduce the problems mentioned above. The polymer choice is also of great importance to avoid an accelerated release of the compound [75]. Electrospun nanofibers could avoid the burst release phenomena observed in nanospheres or nanocapsules, facilitating the controlled release of pesticides. For example, thiram pesticide has been incorporated into polycaprolactone nanofibers by Pouladchang et al. [76], with a pesticide loaded of 26.4–163.4 mg/g; also, Roshani et al. [77] encapsulated thiram pesticide into poly(L-lactic acid) nanofibers with loading efficiency between 50 and 60%, both manufactured by electrospinning technique and with diameters of 29-481 nm and 150-260 nm, respectively. On the other side, essential oils have been used as alternative synthetic pesticides for pest management of foodstuffs. For instance, Allahvaisi et al. [78] evaluated the release of essential oils of Mentha piperita L. and Salvia officinalis L. from poly(lactic acid) nanofibers for fumigant toxicity against first instar larvae of Plodia interpunctella. Pure essential oils completely lost their insecticidal activity after 14 days, whereas at the same period, essential oils in nanofibers had an average of 93% mortality when applied against P. interpunctella.

In addition, nanofibers have been used as pesticides extraction or detection systems. In this context, Komal et al. [79] incorporated cadmium sulfide nanoparticles into the biomass-derived silanized cellulose nanofiber matrix for adsorptive detoxification of pesticide. The nanofibers were derived from sugarcane bagasse, and the nanocomposite demonstrated improved adsorption properties to abolish pesticide contaminants from wastewater. In the same way, Chamuah et al. [80] developed PVA nanofibers but coated with gold nanoparticles to detect deltamethrin, quinalphos, and thiacloprid. The nanofiber substrate, which turned out to be a low-cost, relatively reproducible, and sensitive substrate, can easily estimate the concentration of the pesticide samples below the permissible limit.

On the other hand, Jafari et al. [81] prepared carbon-silica hybrid nanofibers for extracting organophosphorus pesticides, malathion, and chlorpyrifos. The fibers were prepared by carbonizing sol-gel based on electrospun polyacrylonitrile and tetraethyl orthosilicate nanofibers as carbon and silica precursors. The extraction processes were affected by the spinning distance, voltage, feeding rate, stirring rate, salt concentration, temperature, and extraction time. The nanofibers presented relative recoveries of the proposed method in the range of 80–111% for actual samples. Moreover, introducing tetraethoxysilane into the polyacrylonitrile solution increased the specific surface area, enhancing extraction efficiency better than commercial fibers (polydimethylsiloxane).

## 4. Possible Instability Phenomena in Nanopesticides

The stability of colloidal suspensions is a function of their surface energy [82] and does not usually tend to phase separation until a few months after preparation since the settling process is slow for submicrometer particles and is further minimized by Brownian motion. However, particle instability phenomena can occur with time [49]. These usually occur after irreversible processes like coalescence or flocculation or after reversible processes, such as creaming, sedimentation, and agglomeration. Each of these will depend on the density of the dispersed phase, and the strength of the interactions generated that exceed the repulsive energy [82, 83]. Therefore, it is essential to ensure that polymeric nanocarriers remain viable over long storage periods and active in the sputtering environment.

Several factors affect the colloidal physicochemical stability in NPs. On the one hand, due to the diversity of locations where pesticides are used and how they are transported, the environmental temperature can affect the stability of pesticide formulations, especially after undergoing four seasonal temperature changes in a year [69]. Physical instability is mainly induced by aggregation and sedimentation of particles in the formulation and could be further aggravated by temperature fluctuations [84]. At low temperatures, water-based formulations may freeze or decrease solubility and cause crystallization, damaging the stability of pesticide formulations, while probable thermolysis, sedimentation, and flocculation would also destroy the stable system at high temperatures [69]. Thus, Roque et al. presented a study on the colloidal thermal stability at three different storage temperatures (4, 25, and 37°C) of different polymeric nanoparticles formed by PLA, PLGA, poly(arginine), xanthan gum, alginate, chitosan, and chitosan/dextran. The results revealed that temperature influenced the particle size, with an increase of PDI at higher temperatures with values ranging from 0.169 to 2.344 [85], suggesting perturbation of the system stability due to coalescence between the nanoparticles.

Another crucial point to consider is the adaptability of polymer-based NP formulations to water quality since changes in ionic strength can cause destabilization of the diffuse layer in the electrical double layer of the particles. The association of the particles with the increase in ionic strength is favored due to the decrease in the thickness of the electrical double layer [69, 82, 86]. Similarly, alterations in the pH of the test media can influence the surface potential and thus influence the stability and transport of nanoparticles [86]. Variations in pH values are due to different soil types, the nature of the plant, or as a cause of pests and pathogens; similarly, pH in the digestive system of insects varies [87]. Therefore, both ionic strength and pH have been found to be significant factors that destabilize the water-soluble polymers and polysaccharides that are often used in the construction of NPs [88, 89].

On the other hand, since several of the pesticides used have hydrophobic structures, one of the advantages of polymeric nanopesticides is increasing the dispersion of these active ingredients in aqueous media. In 2022, Zhao et al., to overcome this limiting feature of pesticides, developed polymeric nanocarriers composed of zein and chitosan oligosaccharides covalently connected, which were loaded by a model pesticide avermectin (hydrophobic and light instable). Obtain a decrease in the encapsulation efficiency of the pesticide by increasing the rate of chitosan grafting, which generates an increase in the hydrophilicity of the structure and subsequent agglomeration of the carrier or avermectin, thus destabilizing the NP system [87].

## 5. Stability Strategies for Polymer-Based Nanopesticides

### 5.1. Physicochemical Aspects

The high surface area favors a high surface free energy that promotes the flocculation of NPs. Most NP carriers include a stabilizing agent on the surface that increases the zeta potential and/or modulates steric effects to promote the repulsion of the nanoparticles. The same phenomenon can occur with active substances in nanomaterial form that do not include a carrier. Flocculation and eventually coalescence of NPs can cause a lack of effectiveness of the active substances, increase bioaccumulation, or incur general toxicity events [90].

### 5.2. Stabilizers

Surface active agents (surfactants) decrease surface and interfacial tension while maintaining the stability of dispersed systems. Surfactants can alter the average particle size and the polydispersity index before, during, and after the formation of the NP. Surfactants are the main stabilizers of polymeric nanocarriers, although not the only ones, and they have the modality of having nonionic, cationic, anionic, or amphoteric structures with an overall hydrophilic-lipophilic balance. The nature of the surfactant chosen will depend on the chemical composition of the polymeric carrier, the type of nanopesticide-application surface interface, and the type of application required. Some common stabilizers for polymeric nanocarriers are tween 80, span 80, polyvinyl alcohol, some poloxamer variants, polyethylene glycol, and mixtures of stabilizers [91]. Formulation concentrations can range from 0.1 to 5%. The type of nanopesticide-stabilizer interaction is often noncovalent with a temporary permanence of the stabilizer on the surface. The noncovalent grade nanopesticide-stabilizer type of interaction will preserve limited stability, especially in liquid dispersed systems. The initial deposition of the stabilizer through various adsorption phenomena will follow a desorption pattern until an equilibrium is reached between the concentration of stabilizer dissolved in the liquid medium and that present on the surface of the nanoparticle. Other nanopesticide-stabilizer interaction tools are of the covalent type and can involve a previous chemical reaction between the stabilizer and the polymer before forming the nanoparticle, or once the nanoparticle is formed, the stabilizer can be coupled to the surface. In the first strategy, there is a free space of interaction governed by the type of reaction and the functional groups of the polymer and the stabilizer, and some stabilizer fractions can be included in the nucleus of the nanoparticle and form a kind of vesicles. The second strategy involves obtaining nanoparticles without stabilizers and the subsequent coupling reaction. Another alternative includes the addition of a first stabilizer in a low concentration by adsorption and after the addition by covalent coupling of a second stabilizer of main interest in a higher concentration. Sometimes, the presence of the first stabilizer, even in low concentrations, can block the reaction efficiency in coupling the second and main stabilizer. The stabilizer can be a spacer for the second coupling of a targeting agent in more complex architecture strategies, including amino acids, peptides, proteins, DNA, RNA, siRNA, carbohydrates, antibodies, drugs, and combinations. Eventually, this greater complexity allows a degree of targeting, increasing the efficiency of the NPs. However, the degree of sophistication always affects the cost of the product, and the cost-benefit balance becomes essential in industrial applications.

### 5.3. Additional Excipients

**Figure 2:**
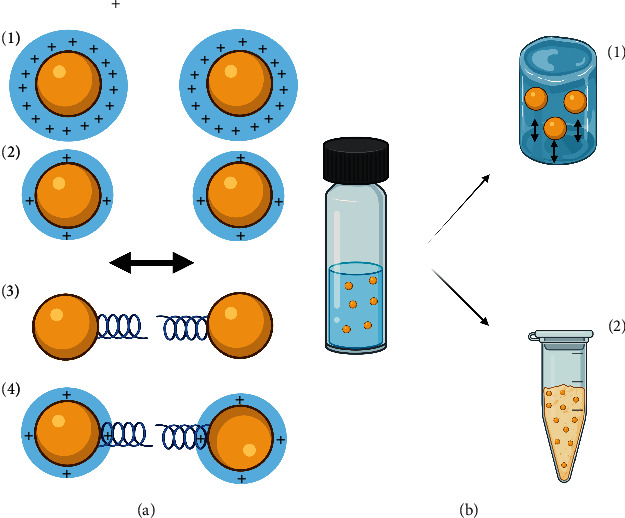
Stability strategies for polymer-based nanopesticides. (a) Physicochemical stabilization: (1) repulsion of particles with high electric charge density, (2) repulsion of particles with low electric charge density, (3) repulsion of particles by steric mechanisms, and (4) repulsion of particles with low electric charge density and steric mechanism. (b) Physical stabilization: (1) increase of the medium's viscosity as a strategy to reduce the sedimentation rate and (2) lyophilization of the product.

The presence of antioxidants, chelators, buffers, and viscous systems can increase the stability of NPs, depending on the type of active substance. In particular, the addition of viscous systems can facilitate the wetting of the NPs, decrease the attraction of the particles, prevent cremation, and delay the sedimentation phenomena (Figure 2). Also, viscosity can function as film formers by creating depot and sustained release systems at the application site.

### 5.4. Lyophilization

The stability of NPs in an aqueous medium could be questionable due to the intrinsic instability of colloidal systems in any application. There are formulations of nanomaterials that demonstrate long suspension times and guarantee a shelf life and application by the user of a few months or just over a year. However, in the case of nanomaterials of the active substance, it is possible to record the formation of sediments or cremation with an irreversible change in size, polydispersity index, zeta potential, and morphology [92], while for polymeric nanocarriers, there may be the possibility of erosion and degradation of the polymeric matrix during storage in aqueous dispersion, with the subsequent anticipated release of the active substance. The result, in either case, is a loss of production efficiency. The solid state as a powder for resuspension is one of nanomaterials' most convenient commercial presentation strategies. The solid-state presentation could require a lyophilization process in most examples of polymer-based nanopesticides. Lyophilization consists of a sublimation process to remove water under reduced pressure with a supply of energy in low-temperature ranges, facilitating the product's drying without altering the product's structural integrity [93]. The lyophilization processes of polymer-based nanopesticides can produce the coalescence of the polymeric nanoparticles irreversible changes in size and shape of the nanocarriers, for which the presence of cryoprotective agents is necessary [93]. Traditionally, the addition of cryoprotective sugars such as trehalose, maltose, sucrose, and mannitol has adequate performance in a concentration range of 2 to 5% in preserving the physical properties of the nanoparticles [94].

## 6. Tools for Monitoring the Stability of Polymeric Nanopesticides

**Figure 3:**
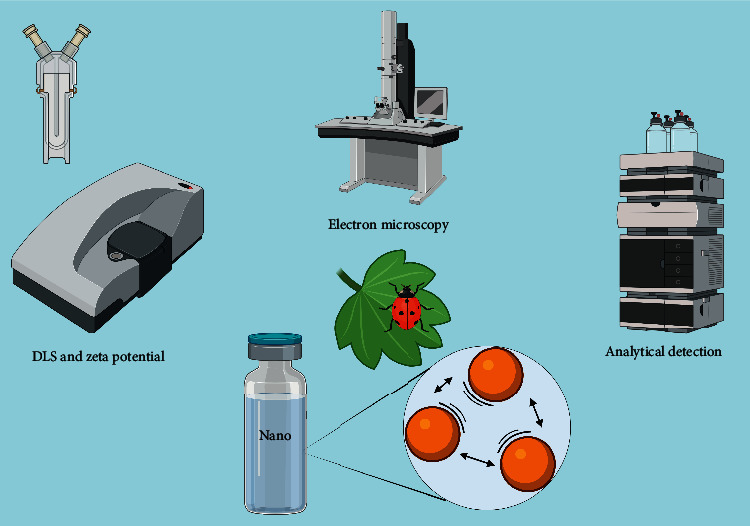
Monitoring of the stability of polymeric nanopesticides. In chronological sequence: determination of particle size and zeta potential, morphological analysis, and analytical characterization.

Polymeric nanomaterials have a high surface energy that favors flocculation phenomena to reduce their exposure area. Coalescence is the posterior and irreversible phenomenon that involves the loss of the original shape and size [95]. The absence of nanometric characteristics changes the effectiveness of polymeric nanopesticides mainly in controlled release systems, vectorized, and in the absorption and permeation capacity. The usual strategy for monitoring the stability of polymeric nanopesticides is the correlation of visual inspection, particle size, zeta potential values, and morphology using electron microscopy [96] (Figure 3).

### 6.1. Dynamic Light Scattering (DLS)

The DLS technique is traditionally the quality control tool of the first choice in most polymeric nanomaterial's characterization studies. DLS is the primary tool for monitoring the stability of nanomaterials due to the fast analysis time, reproducibility, robustness, low cost, and representative nanoparticle registration. Additionally, a vast database of DLS records can be compared with average particle size or PDI values. Although there are different companies, most of the literature studies are supported on Malvern Panalytical platforms. Most indicative of instability phenomena denote an increase in particle size and PDI values; however, the magnitude of the change depends on the composition and complexity of the polymeric nanopesticides. Therefore, it is convenient to establish adequate confidence intervals for each situation to ensure the product's effectiveness. Sometimes, an increase in particle size and PDI in specific ranges does not detract from effectiveness.

### 6.2. Zeta Potential

A zeta potential measurement is an analysis tool that requires more time than the DLS and is usually less reproducible, robust, and slightly higher analysis cost. The zeta potential measurement is based on an electrophoretic shift of the NPs and is an indirect determination through the Henry equation. The zeta potential measurement estimates the electric charge density in the slipping plane and directly predicts NP stabilization by a similar charge repulsion mechanism. Some typical values for NPs are >|20 mV|, and vice versa, values lower and close to 0 mV indicate low stability of dispersed systems in water. The recording methodology and the interpretation must be cautious because the electrophoretic movement can be affected by multiple factors, and not all NPs are stabilized by a repulsion mechanism of similar charges. Steric stabilization mechanisms usually decrease the zeta potential to low values while the dispersion remains stable; there may also be a steric stabilization mechanism associated with a contribution from the electrical density of the slipping plane. The instability phenomena are associated with the desorption processes of the stabilizing agents and consequently the reduction of the zeta potential or in nanoparticle flocculation phenomena with the reduction of the surface area.

### 6.3. Morphology

Adequate monitoring of the stability of NPs usually involves the correlation of the determination of the particle size, the PDI value, measurement of the zeta potential, and the characterization of the morphology. According to the type of architecture of the polymeric nanopesticides, the morphology characterization tools can include scanning electron microscopy, transmission electron microscopy, or atomic force microscopy. Other variants of microscopy are valid as long as they allow adequate resolution in the nanometric size range. Solid architectures are preferably visualized in SEM, while TEM is suitable for lax structures. SEM allows 3D appraisals, while AFM is characterized by higher resolution and easy sample processing [97]. It is always convenient to consider that in SEM and TEM, the processing of the sample can result in a modification of the architecture and particle size. It is common to have more minor particle size records by SEM and TEM than by DLS.

### 6.4. Identity

Analytical characterization tools are suitable for monitoring labile NPs. One of the objectives of polymeric carriers is to protect the active substance; for this reason, analytical identity tests are essential in monitoring the stability of NPs. Therefore, stability studies include monitoring at different times and temperatures. The preformulation steps also include identity studies in the interaction between the different nanopesticide materials to prevent possible incompatibility and degradation reactions. The most used strategies include chromatographic techniques coupled to mass spectrometry detection, such as liquid chromatography with mass spectrometry, liquid chromatography with diode array detector, and inductively coupled plasma mass spectrometry [96].

## 7. Environmental Risks of Polymer-Based Nanopesticides

**Figure 4:**
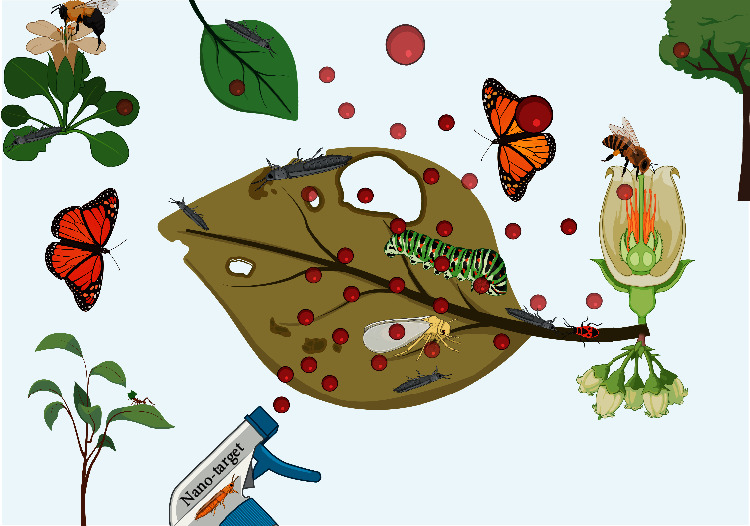
Nanopesticides are encouraged to be selective and efficient and with less impact on the environment.

Among the different carrier systems, polymeric nanoparticles have been studied for agricultural purposes due to their solid matrices, which protect the bioactive compound from degradation [98, 99]. Although carrier systems may offer a range of benefits, they are still in the early developmental stage (Figure 4), and their risk to human, animal, plant, and environmental health is not yet fully understood. Numerous researches assessing the toxicity of engineered nanomaterials to agricultural plant species have been done; however, much of them have focused on short-term, high-dose exposure scenarios often conducted in model media [6]. Unlike conventional pesticides, the uptake, bioavailability, and toxicity of NPs depend on the particle number concentration, particle size distribution, and the ratio of “free” and nanoparticle-bound active ingredients [100].

The pesticide fate and behavior during and after its application in the receiving environment are crucial determinants for its potential impact on ecosystems and human health [5]. However, direct measurements of how slow-release nanoformulations would perform in the field are not easy to carry out. Therefore, indirect approaches, such as sorption, degradation in soil, photolysis, or kinetics of efficacy, can be used to measure release rates [5]. Gomes et al. [101] reported the effects of a nanoformulation of atrazine to nontarget soil invertebrates via soil exposure. The authors showed that polymeric nanocapsules containing atrazine were more toxic to the soil invertebrate Enchytraeus crypticus (Oligochaeta) as the nontarget organisms, than the respective non-nanopesticide, a commercial formulation of atrazine (Gesaprim®) [101]. Sousa et al. [102] investigated the herbicidal activity of PCL nanocapsules containing atrazine against Amaranthus viridis (slender amaranth) and Bidens pilosa (hairy beggarticks), in comparison with a commercial formulation of atrazine. For both species, treatment with nanoencapsulated formulations led to a greater decrease in the photosystem II activity than the commercial atrazine formulation. For B. pilosa, the nanopesticide decreased the root and shoot growth more effectively than the commercial formulation, leading to a loss of plant biomass [102].

Mohd et al. [103] compared the fate and uptake of bifenthrin in traditional and nanoencapsulated formulations (Nano A and B, obtained from Vive Crop Protection Inc.) in soil-earthworm systems, using the two earthworm species Eisenia fetida and Lumbricus terrestris. It was reported that earthworms in the nanotreatments accumulated approximately 50% more bifenthrin than those in the non-nanotreatments [103]. Besides, most of the bifenthrin taken up was found in the earthworm tissue in the conventional formulation, while the majority resided in the gut in the nanoencapsulated bifenthrin [104]. Fojtová et al. [104] also found that nanoformulation of pesticides (chlorpyrifos and tebuconazole loaded on polymeric and lipid nanocarriers) affect their behavior in soil and the uptake to earthworms (Eisenia fetida) and plants (lettuce Lactuca sativa). These changes seem to depend on the nanocarrier, compound, soil used, and time.

## 8. Future Challenges of Polymer-Based Nanopesticides

Even though polymer-based nanopesticides are at an early stage of development, it is expected that they will help to reduce the indiscriminate use of conventional pesticides and diminish environmental pollution of agricultural production [105]. The main challenge of polymeric NPs is competing with existing formulations in cost and performance [99]. NPs generally offer superior qualities, high potency, economical, and user and environmentally friendly compared with their conventional counterparts and therefore have promising practical applications [106]. The advantages of polymeric nanoparticles include biocompatibility, biodegradability, the ability to modify and functionalize the surface, incorporation of the active agent without any chemical reactions, and the possibility of modulating the degradation and release of the active agent by a selection of the materials used to prepare the nanoparticles [98]. However, almost all polymeric nanocarriers are synthesized in the laboratory in small amounts; hence, it is necessary to establish standard procedures for a particular group of pesticides, which could be scaled up commercially [57].

Polymer-based nanopesticides, as for any other regulated product, have to demonstrate their safe use without posing potential risks to the consumer and the environment [107]. Therefore, further studies have to be done to understand the compatibility between the pesticides and encapsulation materials and the encapsulation mechanism of pesticide formulations [99]. Characterization data are fundamental to relate the novel qualities of the products to their physicochemical properties, understand the mechanisms involved, and evaluate if the benefits are preserved across a range of agronomic conditions [5]. Smart delivery of pesticides and growth regulators is possible by developing nanodevices [108], including nanosensors for real-time monitoring of soil conditions, crop growth, and pest and disease attack.

Another important challenge in using NPs is their regulation, which should be improved and applied in all countries. In 2015, the European Union and Switzerland were detected as the only world region where nanospecific materials were incorporated in legislation, which included specific information requirements for nanomaterial risk assessment and the obligation to label or report the presence of nanomaterials in products [109]. Changes in legislation can serve as opportunities to foster the development of innovative solutions to maintain or increase crop production while minimizing environmental impact [110].

## 9. Conclusions

During the last decades, pesticides have been key enablers for farmers to produce high-quality and affordable crops by reducing the risk of pathogens and harmful insects. However, its indiscriminate use has caused environmental concerns, calling for the agricultural sector to turn to innovative approaches.

Nanotechnology has arisen as a potential technology for nanopesticides production, offering higher efficacy and lower environmental impact than traditional products. Overall, the reviewed publications in this work revealed an agreement regarding the future potential for novel and more effective NPs formulations. Nevertheless, one major drawback is the limited amount of evidence-based data from real-life applications suggesting that more investigations in field-based conditions need to be carried out to understand the environmental and economic benefits. Importantly, toxicity concerns need to be addressed using standardized methods for assessment.

## Figures and Tables

**Table 1:** Different nanoformulations based on natural and synthetic polymers.

Nature of polymer	Type of polymer	Nanodesign	Active molecule	Target	Ref
Natural	Chitosan-porous carbon NPs	Nanoparticles	Paraquat	Cynodon dactylon	[21]
	Chitosan-lanthanum	Nanoparticles	Avermectin	Magnaporthe grisea	[8]
	Chitosan-alginate Chitosan/tripolyphosphate	Nanoparticles	Imazapyr and Imazapyc	Bidens pilosa	[25]
	Chitosan/tripolyphosphate	Nanoparticles	Paraquat	Photosystem I of spinach leaf tissue	[19]
	Chitosan and cashew gum	Nanogel	Lippia sidoides essential oil	Larvae of aegypti	[42]
	Alginate	Nanohydrogels	Dicamba	—	[26]
	Sodium alginate	Nanocapsules	Pyridalyl	Larvae of Helicoverpa armigera	[43]
	Carboxymethyl cellulose-rosin	Nanocapsules	Avermectin	Plutella xylostella	[44]
	Cellulose	Nanocrystals	Thiamethoxam	Phenacoccus solenopsis	[31]
	Carboxymethyl cellulose-diallyldimethylammonium chloride-Zein	Nanocapsules	Avermectin	Larvae of diamondback moth	[45]
Synthetic	PCL	Nanocapsules	Ametryn, atrazine, and simazine		[33]
	PCL	Nanocapsules	Pretilachlor	Echinochloa crus-galli	[13]
	PLA	Microcapsules	Lambda-cyhalothrin	Plutella xylostella	[35]
	PEG-dimethyl esters	Nanomicelles	Carbofuran	—	[41]
	PEG	Nanocapsules	Clofentezine	Tetranychus urticae	[38]
	PEG-lignin	Capsules	Metribuzin	—	[40]
	PEG-chitosan	Nanospheres	Geranium maculatum and Citrus bergamia essential oils	Culex pipiens	[46]

## Data Availability

The data used to support the findings of this study are available from the corresponding authors upon request.
